# Epidemiology of respiratory infectious diseases after the relaxation of COVID-19 policies: a retrospective study in Xiamen, China

**DOI:** 10.3389/fpubh.2026.1759146

**Published:** 2026-04-29

**Authors:** Linlin Qu, Xiaojun Jing, Ruopei Wang, Kai Zhang, Meiying Su, Lanlan Lian, Chunhua Yang, Houzhao Wang

**Affiliations:** 1 Department of Clinical Laboratory, Xiang’an Hospital of Xiamen University, School of Medicine, Xiamen University, Xiamen, Fujian, China; 2 Department of Equipment and Materials, Xiang’an Hospital of Xiamen University, School of Medicine, Xiamen University, Xiamen, Fujian, China; 3 School of Medicine, Xiamen University, Xiamen, China; 4 Department of Infectious Diseases and Hepatology, Xiang’an Hospital of Xiamen University, School of Medicine, Xiamen University, Xiamen, Fujian, China

**Keywords:** epidemiological characteristics, post-COVID-19 era, respiratory infectious diseases, respiratory pathogens, retrospective study, Xiamen

## Abstract

**Objective:**

This study aimed to analyze the epidemiological patterns of respiratory infectious diseases in Xiamen, Fujian Province, over the year following the relaxation of China’s COVID-19 control measures.

**Methods:**

We conducted a comprehensive statistical analysis of testing data for the six prevalent respiratory pathogens, utilizing throat swab antigen tests, PCR assays, and serological IgM testing. For *Mycoplasma pneumoniae*, diagnosis relied on serological IgM or rapid PCR testing, with PCR results prioritized when both methods were applied. Influenza A/B viruses were identified through throat swab antigen or PCR testing, again favoring PCR outcomes. Respiratory syncytial virus, rhinovirus, and adenovirus were exclusively detected via PCR.

**Results:**

Influenza A virus emerged as the most prevalent pathogen (32.5%), followed by *Mycoplasma pneumoniae* (18.0%). Seasonal trends varied: influenza A peaked in spring, while *M. pneumoniae* surged in autumn and winter. Age-wise, respiratory syncytial virus (RSV) and human rhinovirus predominate in the 0–4 age group; other respiratory viruses predominate in the 5–14 age group; though influenza A and B viruses show relatively high positivity rates in the 15–44 age group. Notably, significant gender disparities were observed across several pathogens (*p* < 0.05).

**Conclusion:**

This study provides a detailed epidemiological snapshot of post-pandemic respiratory pathogens in Xiamen, highlighting distinct age-specific vulnerabilities and seasonal patterns. These findings offer practical guidance for developing targeted, stage-specific intervention strategies to mitigate disease burden in the post-pandemic era.

## 1 Introduction

Respiratory infectious diseases (RIDs), caused by pathogens invading the respiratory tract via droplets, aerosols, and close contact, are characterized by rapid transmission and high outbreak potential (1). Over 200 pathogens have been identified as causative agents of RIDs, including influenza viruses, *Mycoplasma pneumoniae* (MP), rhinoviruses (RhV), adenoviruses (ADV), and respiratory syncytial virus (RSV), among others (2, 3). The substantial disease burden imposed by RIDs underscores the critical need for region-specific epidemiological surveillance and targeted prevention strategies (4).

From 2019 to 2022, China took tough non-pharmaceutical steps to fight COVID-19 (5–7). On January 8, 2023, China made a significant policy shift by discontinuing the Class A infectious disease control measures for COVID-19, as outlined in the Law of the China on the Prevention and Control of Infectious Diseases. Consequently, COVID-19 was reclassified, no longer falling under the category of quarantinable infectious diseases as per the Frontier Health and Quarantine Law. This change has the potential to reshape the pathogen landscape of respiratory infectious diseases. Globally, the post-pandemic era has witnessed an unprecedented rise in respiratory illnesses, even during traditionally low-incidence periods (8–11). This phenomenon, attributed to “immunity debt” (12–14) resulting from reduced germ exposure, has been observed in various countries. For instance, Australia had an odd RSV rise in winter 2020 (15), while the United States reported record-high human metapneumovirus infections in spring 2023 (16). Despite these global trends, the specific impact on southern China remains ambiguous, warranting further investigation,.

In Southern China, regions like Xiamen—a bustling economic hub with high population mobility—are at heightened risk for respiratory viruses, including influenza and SARS-CoV. While previous studies have analyzed RID patterns in Xiamen, they relied on pre- or early-pandemic data, leaving a gap in understanding post-pandemic dynamics following policy easing.

This study addresses a research gap by examining the 2023 epidemiological patterns of six common respiratory pathogens in Xiamen post-COVID-19, emphasizing seasonality, age, and gender trends.

## 2 Methods

### 2.1 Study design and setting

The study focused on patients who took at least one of six pathogen tests between January 1 and December 31, 2023. To be included, patients had to present with at least one respiratory infection symptom, such as fever (≥37.5 °C), rhinorrhea, cough, or sore throat, and have complete test data. Patients were excluded: (1) missing key clinical data (demographics, primary diagnosis, sampling date); (2) test results that could not be traced back to original records; (3) untraceable test results. The analysis was conducted at the patient level, for multiple visits within a short period, each patient was counted once: positive if any test was positive, otherwise negative. To determine a pathogen’s positivity rate, divide the count of patients testing positive for it by the total tested for that pathogen. Positivity was defined as follows: influenza A and B viruses: positive by either PCR or rapid antigen test; respiratory syncytial virus, human rhinovirus, and adenovirus: positive by PCR only (Ct ≤ 35); *mycoplasma pneumoniae*: positive by either serological IgM detection or PCR.

For age stratification analysis, patients were categorized into the following age groups: ≤4 years, 5–14 years, 15–44 years, 45–64 years, and ≥65 years.

### 2.2 Sample collection and laboratory processing

Respiratory samples (throat, nasal, or nasopharyngeal swabs) and EDTA-anticoagulated whole blood were collected from patients. Samples were sent to the lab within 1 h, or stored at 2–8 °C and tested within 24 h.

#### 2.2.1 PCR-based detection

To detect pathogens like influenza A and B viruses, respiratory syncytial virus, and human rhinovirus were identified using a fluorescence PCR probe kit from Shengxiang Biotechnology Co., Ltd., Hunan, China. Following the manufacturer’s guidelines, viral DNA and RNA were isolated from 50 μL respiratory samples. The viral RNA then underwent reverse transcription using the RevertAid First Strand cDNA Synthesis Kit from Thermo Fisher Scientific. The reaction took place in a 25 μL volume, containing specific reaction mixes and primers, under defined temperature conditions: 25 °C for 5 min, 42 °C for 60 min, and 70 °C for 5 min. Fluorescence signals were captured after each extension, with a cycle threshold (Ct) value ≤35 indicating a positive result. The synthesized cDNA was stored at −20 °C for subsequent analysis.

#### 2.2.2 Influenza A/B antigen detection and *M. pneumoniae* IgM serology

In addition to PCR, we used Abbott’s rapid antigen tests (colloidal gold immunochromatography) for influenza A/B via throat or nasopharyngeal swabs, yielding results in 15–20 min. The same method was applied to detect *Mycoplasma pneumoniae* IgM in EDTA blood samples, with similar rapid outcomes.

### 2.3 Quality control

In PCR assays, each run included: Negative control (nuclease-free water); Positive control (plasmid containing target gene sequences); Internal control (human RNase P gene) to monitor sample adequacy and extraction efficiency. For antigen and serological tests, built-in controls were applied as per instructions. Every test cassette has an internal line; its presence ensures valid results.

The lab consistently took part in the EQA program held by NCCL in Beijing, China, and all results were satisfactory throughout the study.

### 2.4 Statistical analysis

SPSS 27.0 was employed statistical analysis. Categorical variables were shown as frequencies and percentages. Chi-square or Fisher’s exact test was used to compare positivity rates across groups.

When comparing positivity rates pairwise among six respiratory pathogens, the Bonferroni correction was used to account for multiple comparisons. With 15 such comparisons, the significance threshold was set at *α* = 0.0033. For other analyses, a two-sided *p* < 0.05 was significant.

## 3 Results

### 3.1 Overall prevalence of respiratory pathogens

**Figure 1:**
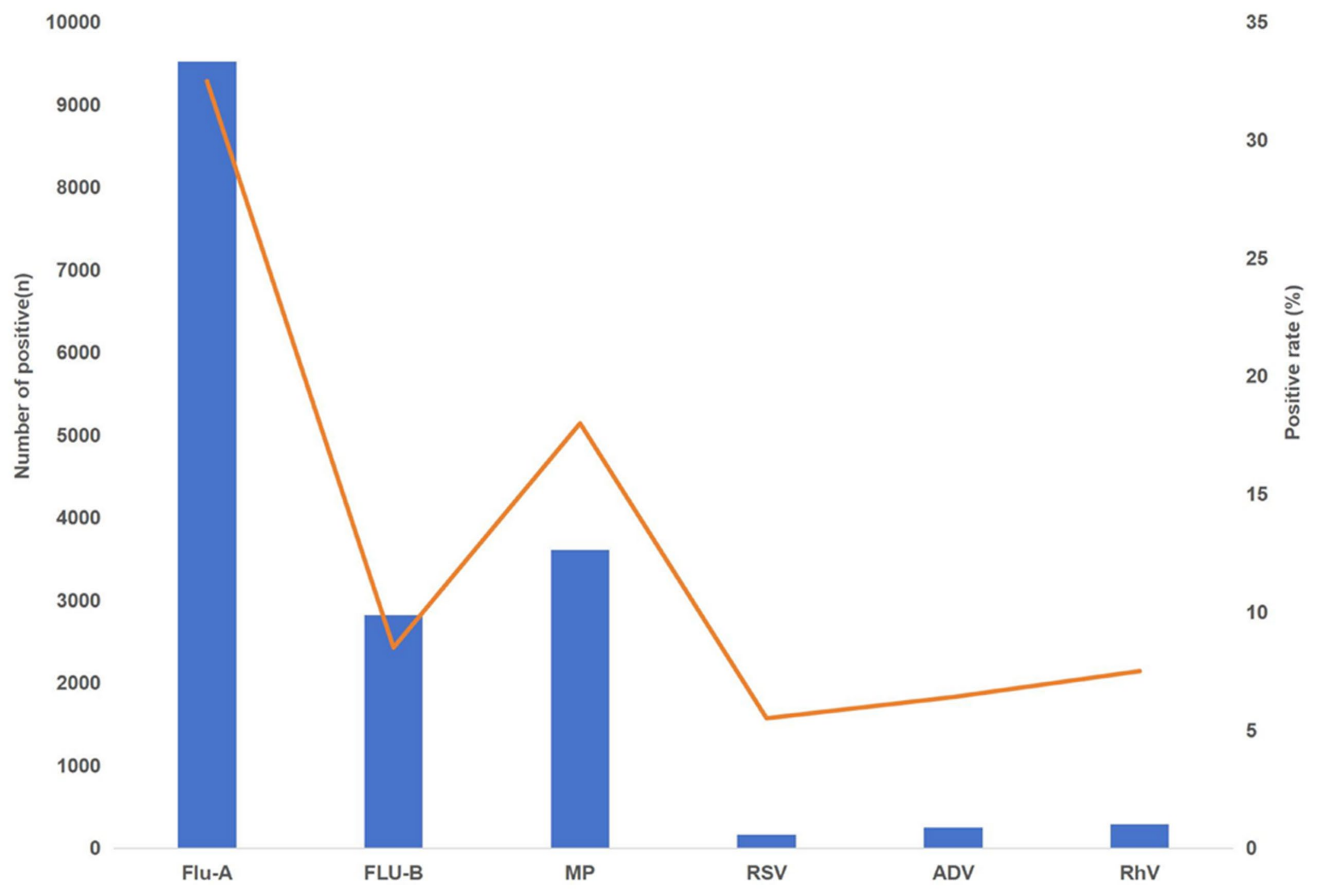
Positivity rates of six pathogens in 2023.

Among six respiratory pathogens tested, influenza A virus and *Mycoplasma pneumoniae* (MP) stood out with notably high positivity rates, at 32.5 and 18.0% respectively, as shown in Figure 1 and Table 1. Statistical analysis via a Chi-square test confirmed marked differences in positivity rates among these pathogens (*χ*^2^ = 7147.917, *p* < 0.001).

**Table 1:** Positivity rates of six pathogen in 2023.

Group	Positive /total (*n*/*N*)	Positivity rate (%)	*χ* ^2^	*p*-value
Influenza A virus (Flu-A)	9526/29318	32.5%		
Influenza B virus (Flu-B)	2817/33009	8.5%		
*Mycoplasma pneumoniae* (MP)	3612/20093	18.0%		
Respiratory syncytial virus (RSV)	160/2915	5.5%		
Adenovirus (ADV)	247/3838	6.4%		
Human rhinovirus (RhV)	289/3841	7.5%		
Over all	16,651/93014	17.9%	7147.92	<0.001

The Chi-square test compared the overall difference in positivity rates among the six pathogens (*χ*^2^ = 7147.92, d*f* = 5, *p* < 0.001). Statistical significance was set at *p* < 0.05. To determine which specific pathogen pairs differ significantly, further post-hoc pairwise comparisons (e.g., with Bonferroni correction) are recommended. See Supplementary Table S1 for details.

Pairwise comparisons showed that influenza A showed significantly higher positivity rates than all other five pathogens (all *p* < 0.001). *Mycoplasma pneumoniae* also demonstrated significantly higher positivity rates than influenza B, RSV, adenovirus, and human rhinovirus (all *p* < 0.001). However, no statistically significant differences were observed between influenza B and human rhinovirus, between RSV and adenovirus, or between adenovirus and human rhinovirus after Bonferroni adjustment (all *p* ≥ 0.495). All other pairwise comparisons were statistically significant (*p* < 0.001). Detailed results of all 15 pairwise comparisons are presented in Supplementary Table S1.

### 3.2 Seasonal distribution of respiratory pathogens

All six respiratory pathogens displayed notable seasonal changes (all *p* < 0.001), with chi-square values ranging from 22.130 to 2000.217. Influenza B virus and influenza A virus exhibited the strongest seasonal specificity, while adenovirus showed the weakest but still significant variation.

**Figure 2:**
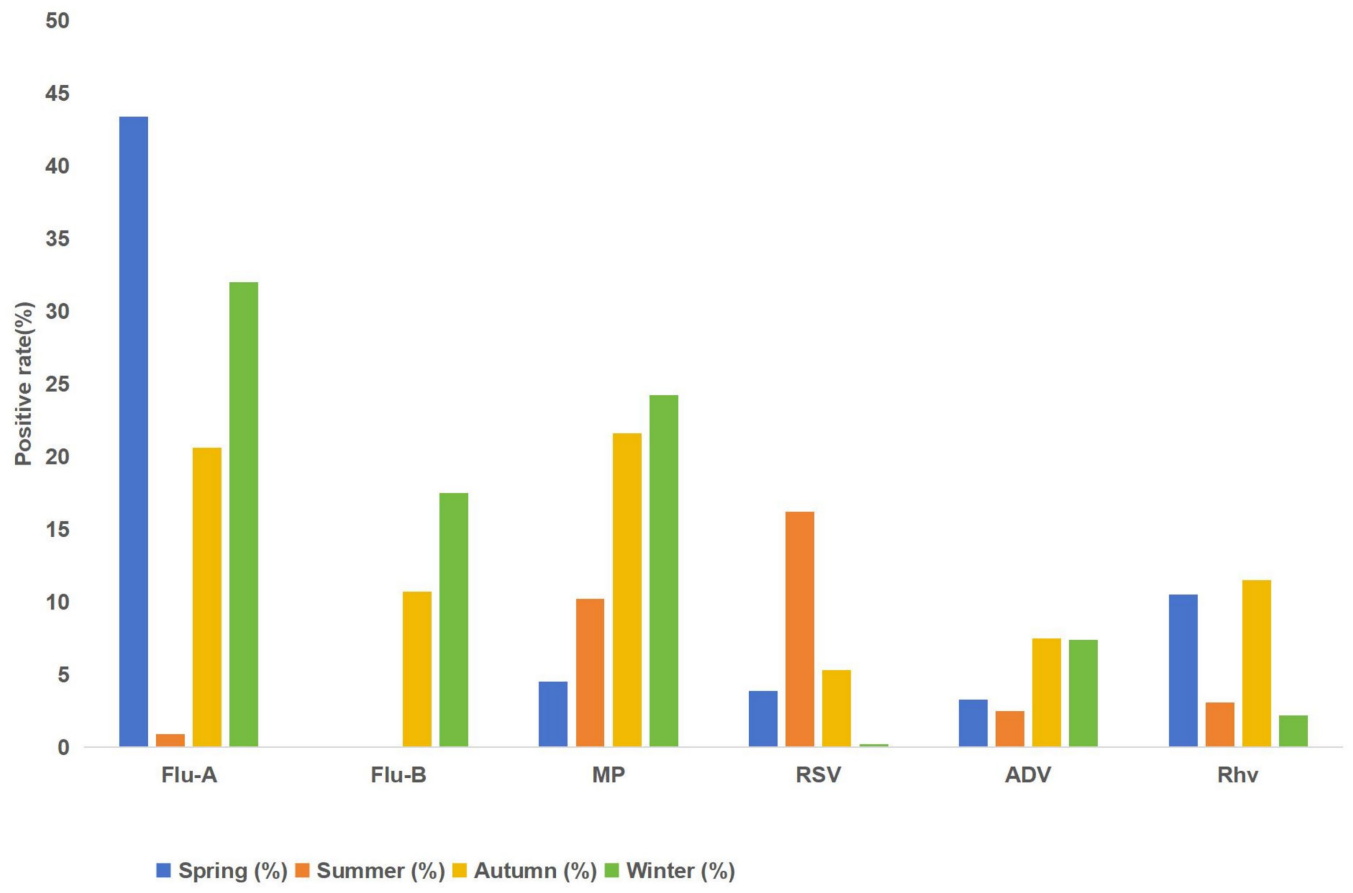
Seasonal distribution of positivity rates for six respiratory pathogens.

Based on their seasonal patterns, pathogens were categorized into four distinct groups (as shown in Figure 2 and Table 2). Influenza A virus follows a spring–winter bimodal pattern, hitting a notable low in summer. Influenza B virus peaks in winter and autumn. *Mycoplasma pneumoniae* is most prevalent in autumn, notably absent in winter. RSV stands out with a summer peak. Adenovirus is present steadily throughout the year, whereas human rhinovirus shows a bimodal peaks in spring and autumn.

**Table 2:** Seasonal distribution of positivity rates for six respiratory pathogens.

Group	Spring (%)	Summer (%)	Autumn (%)	Winter (%)	*χ* ^2^	*p*-value
Influenza A virus	4873/11219 (43.4%)	9/998 (0.9%)	1502/7293 (20.6%)	3142/9808 (32.0%)	1583.09	<0.001
Influenza B virus	3/12350 (<0.1%)	1/1149 (<0.1%)	943/8795 (10.7%)	1870/10715 (17.5%)	2396.47	<0.001
*M. pneumoniae*	135/2968 (4.5%)	361/3529 (10.2%)	1497/6925 (21.6%)	1619/6671 (24.2%)	761.40	<0.001
RSV	14/362 (3.9%)	79/488 (16.2%)	65/1233 (5.3%)	2/832 (0.2%)	153.83	<0.001
Adenovirus	12/362 (3.3%)	12/480 (2.5%)	137/1835 (7.5%)	86/1161 (7.4%)	23.26	<0.001
Human rhinovirus	38/362 (10.5%)	15/480 (3.1%)	211/1839 (11.5%)	25/1160 (2.2%)	107.24	<0.001

Data are presented as number positive/number tested (%). Chi-square tests compared seasonal positivity rates for each pathogen (d*f* = 3). All *p* < 0.001, Statistical significance was set at *p* < 0.05.

### 3.3 Sex differences in pathogen positivity rates

**Figure 3:**
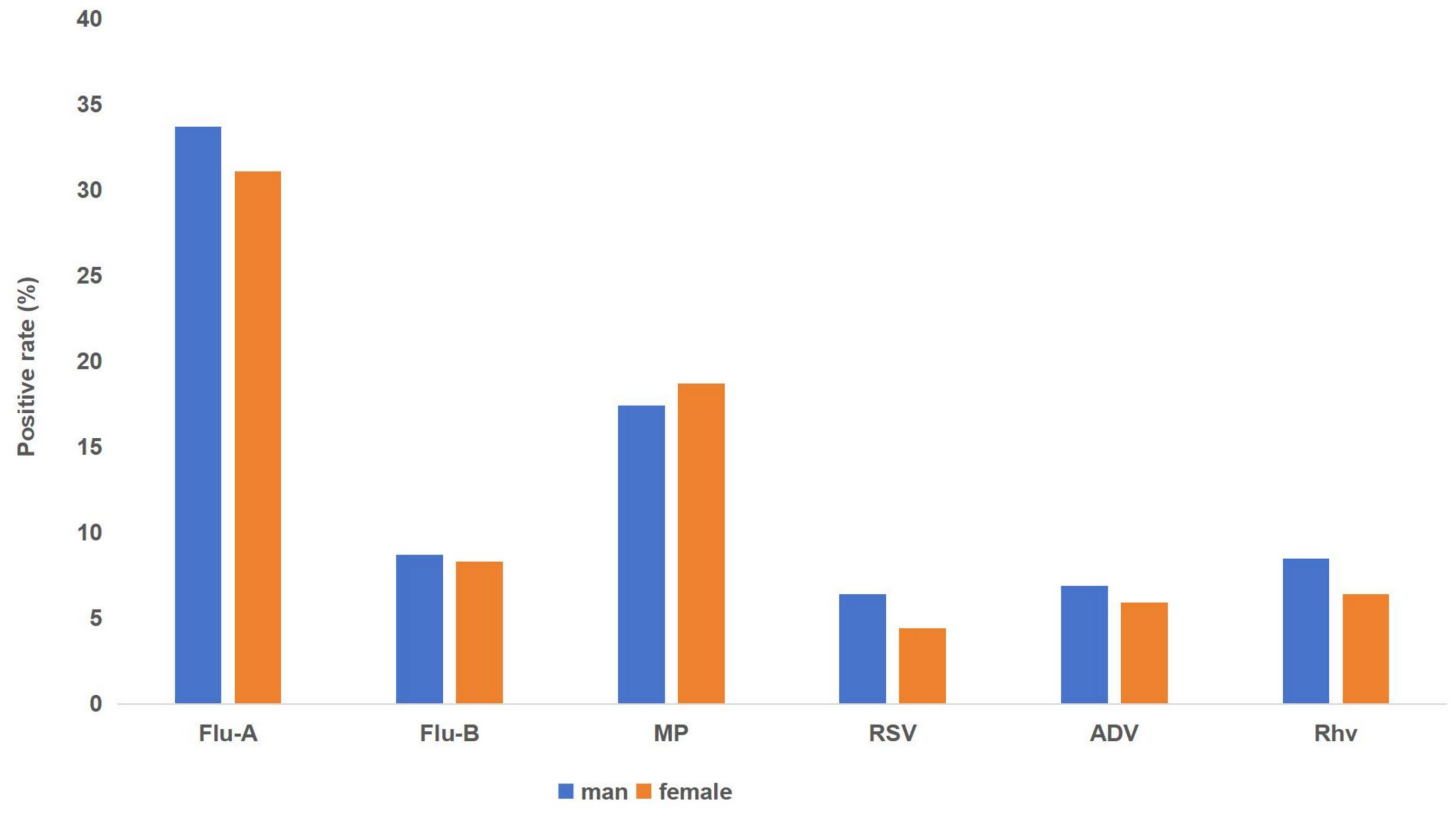
Sex differences in positivity rates of six respiratory pathogens.

Among the six respiratory pathogens, notable gender disparities in positivity rates emerged for four, while two remained unaffected. Specifically, males exhibited a significantly higher susceptibility to Influenza A virus, with a marked statistical difference (*χ*^2^ = 21.714, *p* < 0.01). Similarly, Respiratory syncytial virus (RSV), *Mycoplasma pneumoniae* and human rhinovirus (RhV) also showed significantly higher rates in males (*χ*^2^ values ranging from 5.2 to 6.544, *p*-values between 0.011 and 0.023). In contrast, Influenza B virus and Adenovirus showed no significant gender-based variations in positivity rates (*χ*^2^ values below 2, *p*-values above 0.17). The findings are visually represented in Figure 3 and Table 3.

**Table 3:** Sex differences in positivity rates of six respiratory pathogens.

Group	Male (%)	Female (%)	*χ* ^2^	*p*-value
Influenza A virus	5239/15550 (33.7%)	4287/9481 (31.1%)	21.71	<0.001***
Influenza B virus	1536/17630 (8.7%)	1281/15379 (8.3%)	1.54	0.214
*M. pneumoniae*	1883/10849 (17.4%)	1729/9244 (18.7%)	6.15	0.013*
RSV	99/1544 (6.4%)	61/1371 (4.4%)	5.20	0.023*
Adenovirus	143/2061 (6.9%)	104/1777 (5.9%)	1.87	0.172
Human rhinovirus	176/2062 (8.5%)	113/1779 (6.4%)	6.54	0.011*

Data are presented as number positive/number tested (%). Chi-square tests were used to compare positivity rates between males and females for each pathogen (d*f* = 1). ^***^p < 0.001, ^*^p < 0.05.

### 3.4 Age-stratified epidemiological distribution of respiratory pathogens

**Figure 4:**
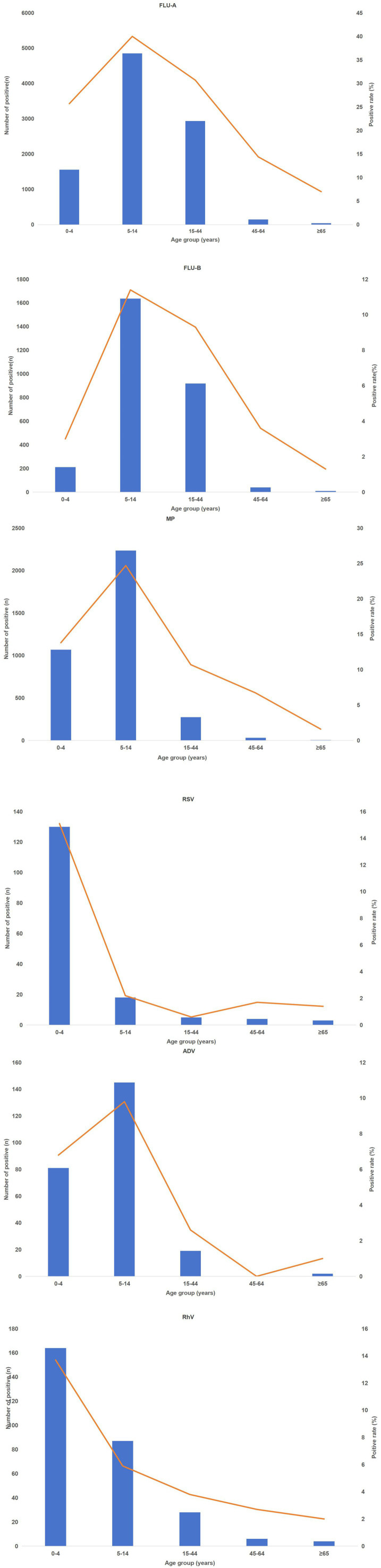
Age distribution of influenza A virus positive cases.

Notable age-based disparities were found across six respiratory pathogens, all statistically significant (*p* < 0.001). Table 4 reveals two main age-related patterns. Respiratory syncytial virus (RSV) and human rhinovirus showed a strong predilection for the youngest age group (0–4 years), with positivity rates of 15.1 and 13.7%, respectively. Meanwhile, Influenza A virus, Influenza B virus, *Mycoplasma pneumoniae*, and adenovirus all peaked in the 5–14 years group, with influenza A virus showing the highest rate (40.0%) across all groups. All pathogens showed the lowest infection rates in people aged 45 and above (see Figures 4).

**Table 4:** Age-specific positivity rates of six respiratory pathogens (%).

Group	0–4 years (%)	5–14 years (%)	15–44 years (%)	45–64 years (%)	≥65 years (%)	*χ* ^2^	*p*-value
Influenza A virus	1555/6050 (25.7%)	4851/12134 (40.0%)	2936/9561 (30.7%)	144/999 (14.4%)	40/574 (7.0%)	770.38	<0.001
Influenza B virus	212/7022 (3.0%)	1637/14332 (11.4%)	919/9845 (9.3%)	40/1102 (3.6%)	9/708 (1.3%)	516.63	<0.001
*M. pneumoniae*	1068/7731 (13.8%)	2236/9043 (24.7%)	273/2562 (10.7%)	30/445 (6.7%)	5/312 (1.6%)	1460.89	<0.001
RSV	130/863 (15.1%)	18/826 (2.2%)	5/784 (0.6%)	4/230 (1.7%)	3/212 (1.4%)	218.54	<0.001
Adenovirus	81/1193 (6.8%)	145/1479 (9.8%)	19/739 (2.6%)	0/222 (0.0%)	2/205 (1.0%)	71.87	<0.001
Human rhinovirus	164/1195 (13.7%)	87/1479 (5.9%)	28/740 (3.8%)	6/222 (2.7%)	4/205 (2.0%)	103.19	<0.001

Data are presented as number positive/number tested (%). Chi-square tests were used to compare positivity rates across age groups for each pathogen (d*f* = 4). All *p* < 0.001, Statistical significance was set at *p* < 0.05.

**Figure 5:**
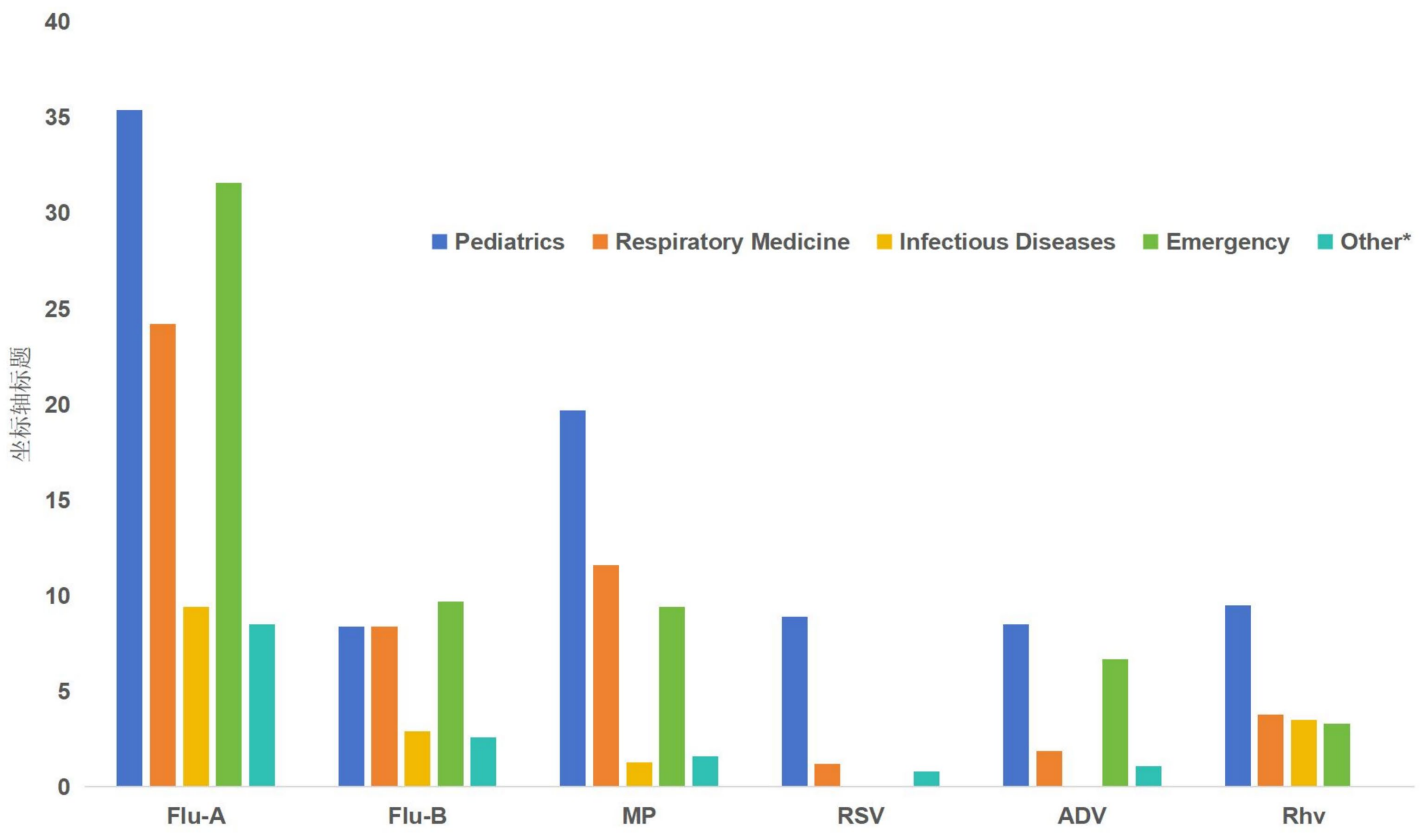
Positivity rates of six respiratory pathogens across clinical departments. Data are presented as positivity rates (%) for each department.

### 3.5 Epidemiological distribution of respiratory pathogens across different clinical departments

**Figure 6:**
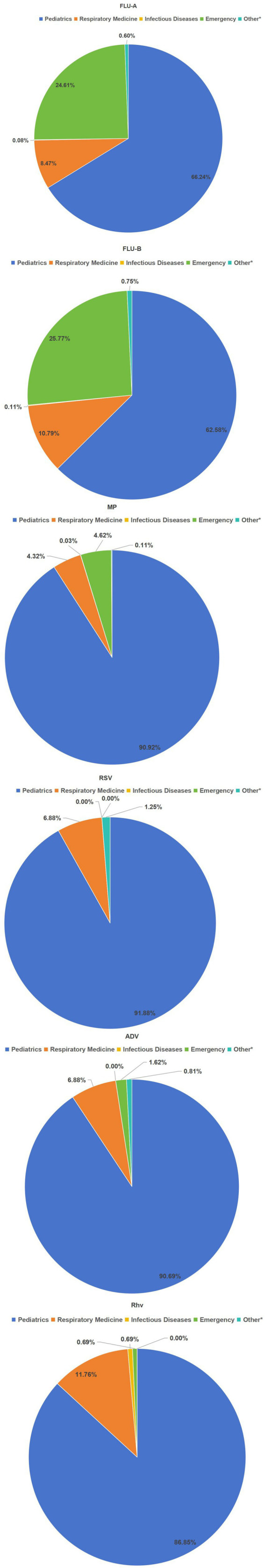
Distribution of six respiratory pathogen cases across clinical departments.

Significant differences in departmental distribution were observed for all six respiratory pathogens (all *p* < 0.01), with chi-square values ranging from 51.538 to 373.744 (Table 5). The influenza A virus was most prevalent in the Pediatrics (35.4%) and Emergency departments (31.6%), with the Respiratory Medicine department following at 24.2%. *Mycoplasma pneumoniae* (MP), respiratory syncytial virus (RSV), adenovirus (ADV), and human rhinovirus (RhV) all exhibited their highest positivity rates in the Pediatrics department (19.7, 8.9, 8.5, and 9.5%, respectively), with markedly lower rates in other departments. Influenza B virus was the most evenly distributed pathogen, with its highest rate in the Emergency department (9.7%). Notably, *M. pneumoniae*, RSV, and adenovirus were not detected in the Infectious Diseases department. Details are shown in Figure 5. These findings indicate that the Pediatrics department is the primary clinical setting for most respiratory pathogens, particularly for MP, RSV, ADV, and RhV. The departmental composition of positive cases for the six respiratory pathogens is shown in Figure 6.

**Table 5:** Positivity rates of six respiratory pathogens across clinical departments (%).

Group	Pediatrics	Respiratory medicine	Infectious diseases	Emergency	Other*	*χ* ^2^	*p*-value
Influenza A virus	6310/17817 (35.4%)	907/3335 (24.2%)	8/85 (9.4%)	2344/7409 (31.6%)	57/672 (8.5%)	373.74	<0.001
Influenza B virus	1763/20966 (8.4%)	304/3623 (8.4%)	3/104 (2.9%)	726/7522 (9.7%)	21/794 (2.6%)	52.09	<0.001
*M. pneumoniae*	3284/16636 (19.7%)	156/1343 (11.6%)	1/80 (1.3%)	167/1778 (9.4%)	4/256 (1.6%)	222.76	<0.001
RSV	147/1649 (8.9%)	11/897 (1.2%)	0/57 (0%)	0/59 (0%)	2/253 (0.8%)	86.22	<0.001
Adenovirus	224/2633 (8.5%)	17/899 (1.9%)	0/57 (0.0%)	4/60 (6.7%)	2/187 (1.1%)	62.61	<0.001
Human rhinovirus	251/2635 (9.5%)	34/900 (3.8%)	2/57 (3.5%)	2/60 (3.3%)	0/189 (0%)	51.54	<0.001

Data are presented as number positive/number tested (%). Chi-square test (d*f* = 4). All *p* < 0.001, Statistical significance was set at *p* < 0.05. *Other departments include but are not limited to ICU, Physical Examination Center, and General Practice.

## 4 Discussion

This study provides a comprehensive epidemiological analysis of six common respiratory pathogens in Xiamen, southern China, in 2023, after COVID-19 restrictions eased. Results show a clear post-pandemic rebound, with FLU-A and MP leading the way, marked seasonal changes from pre-pandemic times, and notable differences in susceptibility by age and sex.

### 4.1 Overall prevalence: FLU-A and MP as the predominant pathogens

In 2023, FLU-A and MP took the lead among pathogens, with positivity rates of 32.5% and 18.0% Their rates far outpaced the other four pathogens (all *p* < 0.001). This shows a notable post-pandemic resurgence and a big change in the local pathogen makeup from the COVID-19 era.

From 2020 to 2022, a Xiamen surveillance study found rhinovirus (29.22%), influenza A virus (19.59%), and RSV (18.36%) to be the top respiratory pathogens, with *Mycoplasma pneumoniae* not in the top five (17). However, after policy relaxation in 2023, FLU-A positivity soared to 32.5%, MP jumped to 18.0% and ranked second and Flu-B rose to 8.50%. Meanwhile, rhinovirus dropped sharply to around 7.5%, showing a notable pathogen shift.

Respiratory pathogens react differently to non-pharmaceutical interventions (NPIs).

FLU-A and MP mainly spread through respiratory droplets and have low basic reproduction numbers, so measures like masks, social distancing, and hand hygiene can greatly curb their spread. However, rhinovirus, RSV, and adenovirus transmit via both droplet and contaminated surfaces, with many asymptomatic or mild cases. This makes NPIs less effective against them, explaining why rhinovirus and RSV stayed common during the pandemic, while FLU-A and MP was largely contained.

The differential susceptibility seen in Xiamen finds further backing from studies in other areas. In Shenzhen, during lockdowns, *Mycoplasma pneumoniae* detection declined by 99.4%, while RSV soared by 118.7% (18). In Taiwan, influenza cases dropped 85.0% during the pandemic, then spiked 619.5% post-restrictions (19). These similar trends across diverse groups confirm NPIs’ pathogen-specific effects.

Secondly, extended suppression led to an “immunity debt”. During the pandemic, the near-total eradication of FLU-A and MP meant that populations, especially the young, missed out on immune-boosting natural infection. Consequently, pre-existing immunity weakened. When NPIs eased in January 2023 and social interactions resumed, many susceptible people encountered these germs, causing the sharp rebound noted in our study, mirroring global trends (20, 21).

These findings demonstrate that COVID-19 NPIs had varying impacts on respiratory pathogens. When restrictions eased, pathogen-specific resurgence driven by immune debt occurred. Xiamen’s “role reversal” between FLU-A/MP and rhinovirus reflects this global post-pandemic trend.

In 2023, rhinovirus and RSV saw a decline, a notable shift from their dominance during the pandemic. This trend can be explained by two interrelated factors. Firstly, a substantial portion of the susceptible population might have already been infected during the pandemic, leaving fewer individuals vulnerable in 2023. Secondly, viral interference may have played a role, with widespread FLU-A and MP possibly suppressing the spread of other respiratory viruses via innate immune responses, a recognized pattern in respiratory virus studies (22).

Findings demonstrate that COVID-19 NPIs had varying impacts on respiratory pathogens. When restrictions eased, pathogen-specific resurgences driven by immune debt occurred. Xiamen’s “role reversal” between FLU-A/MP and rhinovirus/RSV reflects this global post-pandemic trend.

### 4.2 Seasonal distribution: altered epidemiological patterns

Beyond general changes in pathogen prevalence, our 2023 data from Xiamen shows notable shifts in seasonal patterns of several respiratory pathogens versus pre and during pandemic times.

Influenza A virus follows a spring–winter bimodal trend, with a significant summer dip. The spring peak was particularly striking, while the typical summer peak observed during the pandemic period (17) disappeared. This change likely stems from policy easing in January 2023, aligning with the Spring Festival travel rush, boosting spring transmission.

Respiratory syncytial virus (RSV) showed the most dramatic change. Traditionally peaking in winter and spring, RSV became the only pathogen with a summer peak in 2023. This atypical off-season surge is consistent with post-lockdown observations in Australia (23), suggesting that prolonged NPIs can fundamentally disrupt established seasonal rhythms.

In southern China, *Mycoplasma pneumoniae* (MP) typically peaks in autumn-winter. Yet in 2023 (24), it showed an autumn peak only. Pandemic suppression may have shifted its usual winter peak to autumn.

Some pathogens’ patterns remained steady or changed slightly. Influenza B kept winter prevalence, rhinovirus switched to a spring-autumn peak, and adenovirus showed stable, minimal seasonal variation year-round.

The post-pandemic period witnessed not only a resurgence of respiratory pathogens but also profound alterations in their seasonal patterns (25), suggesting a transition from climate-driven to policy-timing-driven seasonality.

### 4.3 Sex differences in respiratory pathogen infections: a pathogen-specific analysis

This study demonstrates that sex differences in respiratory pathogen infections are pathogen-specific. Rhinovirus and RSV showed significantly higher positivity rates in males, which is consistent with multiple studies before and after the pandemic (26, 27). Adenovirus showed no significant sex difference in this study (*p* = 0.172), suggesting that its sex bias may be weak or unstable. For influenza A virus, the direction of sex difference was contradictory: this study and Peer et al. (27) reported higher rates in males, whereas Koirala et al. (26) reported higher rates in females, indicating that the sex bias of influenza A may be significantly influenced by circulating strains, social behaviors, and immune background. *Mycoplasma pneumoniae* showed significantly higher positivity rates in females in both this study and Miao et al. (28). In conclusion, sex is a crucial factor in the epidemiology of respiratory pathogens, and sex-stratified analysis is recommended in future surveillance and prevention strategies.

### 4.4 Age and departmental composition of respiratory pathogen positivity

Post-COVID-19 policy relaxation, Xiamen witnessed a notable shift in the age distribution of respiratory pathogens, with school-aged children (5–14 years) becoming the most affected group. Influenza A virus was most prevalent among them (40.0%), far surpassing adults ≥45 years (14.4%). Similarly, M.pneumoniae was most common in 5–14-years-olds (24.7%), approximately double that in 0–4 years-olds (13.8%); RSV remained concentrated in infants aged 0–4 years (15.1%), with very low proportions in those aged ≥5 years (≤4.5%). All differences were statistically significant (*χ*^2^ tests, all *p* < 0.001).

The “immunity debt” hypothesis offers insight into the observed infection patterns. During the pandemic, non-pharmaceutical interventions (NPIs) limited children’s exposure to common pathogens, causing a buildup of individuals susceptible to infection. Once restrictions were lifted, this susceptible population was exposed en masse, resulting in a “rebound” of infections (29, 30). Serological research reveals that children lose antibodies about seven times faster than adults, with those under five seeing a 15–30% drop in antibody levels during the pandemic (29). Multi-center European studies further demonstrated a clear “dose–response” relationship: diseases with greater declines during the pandemic showed stronger rebounds after relaxation (30). The departmental composition of respiratory pathogen positivity aligned with the findings of the age distribution.

### 4.5 Strengths and limitations

This study has several notable limitations that warrant discussion. Firstly, the absence of comparative data from Xiamen spanning 2019 to 2021 hinders our ability to directly assess shifts in pathogen circulation patterns before and after the pandemic. Secondly, the research was confined to a single hospital over a one-year period, which may restrict the applicability of our findings to broader settings. Thirdly, we did not gather detailed clinical information, such as patient symptoms, vaccination records, or underlying health conditions, making it impossible to evaluate links between infections and clinical outcomes. Additionally, our pathogen detection scope was limited, excluding common pathogens like coronaviruses, *Mycobacterium tuberculosis*, *Streptococcus pneumoniae*, and *Haemophilus influenzae*, and we did not examine co-infections. Future research should broaden pathogen detection, undertake multicenter and long-term studies, and incorporate clinical data for deeper analysis. Finally, positive results here only denote the presence of pathogen nucleic acid or IgM antibodies, not confirmed clinical infections.

## 5 Conclusion

In 2023, the first year after China modified its COVID-19 prevention and control measures, this study explored the epidemiological traits of six prevalent respiratory pathogens in Xiamen. Influenza A virus, *Mycoplasma pneumoniae*, and influenza B virus were the main culprits. The former surged in spring, while RSV dominated summer-autumn. Most pathogens targeted kids, with influenza viruses also notably affecting adults. The findings underscore the need for ongoing vigilance regarding respiratory pathogens’ epidemiological trends post-pandemic, especially influenza A and RSV in off-season periods. This research offers foundational data on Xiamen’s respiratory pathogens after policy shifts.

## Data Availability

The data analyzed in this study is subject to the following licenses/restrictions: throat, nasal or nasopharyngeal swabs and EDTA anticoagulant whole blood samples. Requests to access these datasets should be directed to llqu@xah.xum.edu.cn.

## Supplementary material

SUPPLEMENTARY TABLE S1 Pairwise comparisons of positivity rates among six respiratory pathogens with Bonferroni correction.
