# Supplementary material for: Epidemiology of respiratory infectious diseases after the relaxation of COVID-19 policies: a retrospective study in Xiamen, China
Source: Front Public Health. 2026 Apr 29;14:1759146. doi: 10.3389/fpubh.2026.1759146 (PMC13167966; doi:10.3389/fpubh.2026.1759146)
Supplement: SUPPLEMENTARY TABLE S1 — Pairwise comparisons of positivity rates among six respiratory pathogens with Bonferroni correction. [file Table_1.pdf]

Supplementary Table S1. Pairwise comparisons of positivity rates among six respiratory pathogens with Bonferroni correction

Comparison	χ^2	P value	Adjusted P value*	Significance
Influenza A vs. Influenza B	5611.648	<0.001	<0.001	***
Influenza A vs. *M. pneumoniae*	1286.941	<0.001	<0.001	***
Influenza A vs. RSV	919.748	<0.001	<0.001	***
Influenza A vs. Adenovirus	1108.409	<0.001	<0.001	***
Influenza Avs. Human rhinovirus	1015.965	<0.001	<0.001	***
Influenza Bvs. *M. pneumoniae*	1046.505	<0.001	<0.001	***
Influenza B vs. RSV	32.680	<0.001	<0.001	***
Influenza B vs. Adenovirus	19.858	<0.001	<0.001	***
Influenza B vs. Human rhinovirus	4.547	0.033	0.495	ns
M. pneumoniae vs. RSV	289.622	<0.001	<0.001	***
M. pneumoniae vs. Adenovirus	317.332	<0.001	<0.001	***
M. pneumoniae vs. Human rhinovirus	258.231	<0.001	<0.001	***
RSV vs. Adenovirus	2.622	0.105	1.000	ns
RSV vs. Human rhinovirus	11.064	<0.001	<0.001	***
Adenovirus vs. Human rhinovirus	3.503	0.061	1.000	ns

Note: Data are presented as number positive/number tested (%). Pairwise comparisons were performed using the chi-square test with Bonferroni correction for multiple comparisons (15 comparisons). The corrected significance level was set at $\alpha = 0.05 / 15 = 0.0033$. ***P_raw < 0.001; **P_raw < 0.01; *P_raw < 0.05; ns, not significant (P_raw ≥ 0.0033).
